# Association Between Early and Long-Term Changes in Liver Stiffness in Patients with MASLD Undergoing Serial Magnetic Resonance Elastography

**DOI:** 10.3390/diagnostics16121833

**Published:** 2026-06-13

**Authors:** Shohei Kimura, Yutaka Yasui, Nobuharu Tamaki, Mayu Higuchi, Takuya Shima, Mina Taguchi, Yudai Yamazaki, Risa Seike, Naoki Uchihara, Yuki Tanaka, Ryohei Kobayashi, Junko Yagita, Yuka Kasano, Yasuyuki Komiyama, Kenta Takaura, Hitomi Takada, Shohei Tanaka, Chiaki Maeyashiki, Yuka Takahashi, Hiroyuki Nakanishi, Kaoru Tsuchiya, Namiki Izumi, Masayuki Kurosaki

**Affiliations:** Department of Gastroenterology and Hepatology, Musashino Red Cross Hospital, 1-26-1 Kyonan-cho, Musashino-shi, Tokyo 180-8610, Japan

**Keywords:** MASLD, magnetic resonance elastography, liver stiffness, longitudinal change

## Abstract

**Background/Objectives**: Magnetic resonance elastography (MRE) provides an accurate and reproducible non-invasive assessment of liver stiffness and fibrosis severity in metabolic dysfunction-associated steatotic liver disease (MASLD). Although longitudinal changes in liver stiffness have been associated with clinical outcomes, the association between short-term and longitudinal changes remains unclear. This study aimed to evaluate the association between short-term and long-term changes in MRE-derived liver stiffness in patients with MASLD undergoing serial MRE assessments. **Methods**: In this retrospective cohort study, 100 patients with MASLD who underwent three MRE examinations at approximately baseline, 1 year, and 3 years were analyzed. Early response was defined as a ≥19% reduction in liver stiffness at the second examination relative to baseline. The association between early response and long-term liver stiffness changes was evaluated using logistic regression analysis. **Results**: A total of 22 patients (22%) were classified as early responders. Early responders were significantly more likely to achieve a ≥19% reduction in liver stiffness at the third MRE compared with non-early responders (59% vs. 27%, *p* = 0.006). In multivariable analysis adjusting for baseline liver stiffness, early response remained independently associated with long-term improvement (odds ratio 3.41, 95% CI 1.18–9.86; *p* = 0.023). Higher baseline liver stiffness was also associated with subsequent improvement (OR per 1 kPa increase, 1.44, 95% CI 1.11–1.87; *p* = 0.006). **Conclusions**: Early reductions in liver stiffness measured by MRE were associated with subsequent long-term improvements in patients with MASLD, suggesting that short-term MRE changes may provide insight into subsequent longer-term stiffness changes.

## 1. Introduction

Metabolic dysfunction-associated steatotic liver disease (MASLD) is defined as steatotic liver disease accompanied by at least one cardiometabolic risk factor in the absence of harmful alcohol intake and represents a broad disease spectrum [1]. Fibrosis stage is one of the most important factors for long-term prognosis in patients with MASLD, regardless of the underlying etiology [2,3,4]. Accurate assessment and longitudinal monitoring of liver fibrosis are therefore essential for risk stratification and clinical management in MASLD patients [5].

Although liver biopsy remains the gold standard for fibrosis assessment, its invasiveness, potential complications such as bleeding and pain, limited repeatability, and associated costs restrict its use, particularly for longitudinal evaluation [6,7,8]. Consequently, non-invasive modalities have increasingly been adopted in both clinical practice and research settings [9,10,11]. Among these, magnetic resonance elastography (MRE) enables quantitative assessment of liver stiffness by visualizing propagating mechanical shear waves using MRI and has demonstrated a strong correlation with histological fibrosis stage [12]. Its high diagnostic accuracy and repeatability have led to the increasing adoption of MRE as a non-invasive alternative to liver biopsy, including its incorporation into clinical trials evaluating liver fibrosis [13].

Previous longitudinal studies have demonstrated that changes in liver stiffness measured by MRE are associated with clinical outcomes in patients with chronic liver disease [14,15]. In particular, an increase in liver stiffness over time has been shown to correlate with a higher risk of hepatocellular carcinoma, hepatic decompensation, and all-cause mortality. These findings suggest that dynamic changes in MRE-derived liver stiffness are associated with prognosis and may reflect multiple underlying pathological changes, including fibrosis progression and inflammatory activity. Accordingly, serial assessment of liver stiffness using MRE has attracted increasing attention as a useful non-invasive marker for long-term prognosis.

Despite increasing evidence for the prognostic value of longitudinal MRE changes, most prior studies have assessed liver stiffness over relatively short or variable follow-up periods, and data from repeated MRE examinations beyond two time points remain limited. Moreover, it remains unclear how short-term changes in liver stiffness are associated with subsequent long-term changes in liver stiffness.

Therefore, this study aimed to investigate the association between short-term and long-term changes in MRE-derived liver stiffness in patients with MASLD undergoing serial MRE assessments.

## 2. Methods

### 2.1. Study Design

This retrospective cohort study was conducted at Musashino Red Cross Hospital, a tertiary referral center for liver disease in Tokyo, Japan. A total of 4751 consecutive patients with chronic liver disease who underwent magnetic resonance elastography (MRE) between January 2015 and May 2025 were initially screened. Among these patients, those diagnosed with metabolic dysfunction-associated steatotic liver disease (MASLD) who underwent MRE on 3 or more occasions were identified (*n* = 183).

**Figure 1:**
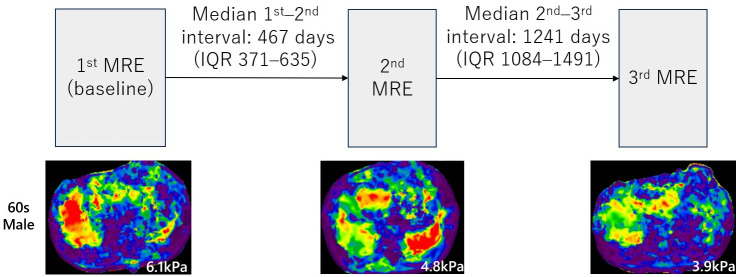
Study design and timing of serial MRE assessments. Liver stiffness was measured at baseline (first MRE), at the second assessment, and at the third assessment. The median interval between the first and second MRE was 467 days (IQR 371–635), and that between the first and third MRE was 1241 days (IQR 1084–1491). The study cohort included patients with MASLD evaluated between January 2015 and May 2025 (*n* = 100). Representative serial MRE images from a patient undergoing three sequential MRE assessments are shown to illustrate longitudinal changes in liver stiffness over time. The color maps represent MRE-derived liver stiffness maps, in which warmer colors generally indicate higher stiffness and cooler colors indicate lower stiffness.

Patients with the second MRE performed approximately 1 year after the first MRE and the third MRE performed approximately 3 years after the first examination were included to standardize follow-up timing (*n* = 113). Patients with an interval of less than 350 days between the second and third MRE examinations were excluded (*n* = 8), as were those with failed MRE acquisition due to technical issues or poor image quality (*n* = 5). The median interval between the first and second MRE examinations was 467 days (IQR 371–635), and the median interval between the first and third examinations was 1241 days (IQR 1084–1491) (Figure 1).

### 2.2. MRE Assessment

MRE was performed using a 1.5T Signa HDxt MRI system (GE Medical Systems, Waukesha, WI, USA) equipped with MR Touch (GE Healthcare, Waukesha, WI, USA). Hepatic shear waves were induced using a passive driver placed lateral to the xiphoid process, operating at a frequency of 60 Hz. Liver stiffness images were obtained using a gradient echo-based sequence, and stiffness maps were reconstructed from wave propagation data [16].

For analysis, regions of interest (ROIs) were manually placed in the right hepatic lobe while avoiding the liver capsule, edges, gallbladder, intrahepatic vessels, bile ducts, focal lesions, and imaging artifacts. The liver stiffness measurement (LSM) was defined as the average value derived from three ROIs.

Baseline MRE-derived fibrosis categories were defined descriptively based on thresholds reported in a previous meta-analysis: F0, <2.61 kPa; F1, 2.61–2.97 kPa; F2, 2.97–3.62 kPa; F3, 3.62–4.69 kPa; and F4, ≥4.69 kPa [11].

### 2.3. Clinical and Laboratory Data

Laboratory parameters, including aspartate aminotransferase (AST), alanine aminotransferase (ALT), γ-glutamyl transpeptidase (GGT), total bilirubin (T-Bil), hemoglobin A1c (HbA1c), total cholesterol (T-chol), and platelet count (PLT), were obtained from routine blood tests performed within 1 month of each MRE examination.

During the study period, metabolic comorbidities associated with MASLD, including hypertension, diabetes mellitus, and dyslipidemia, were managed according to Japanese clinical guidelines. Information about recommended lifestyle and pharmacological treatment was provided to patients in routine practice. Management decisions were made individually by the treating physicians based on clinical needs and patient preferences.

### 2.4. Primary Endpoint

The primary endpoint was to assess whether early improvement in liver stiffness at 1 year was associated with long-term changes in liver stiffness at 3 years. Patients were classified as early responders or non-early responders based on a ≥19% reduction in liver stiffness at 1 year (LSM_2_/LSM_1_ ≤ 0.81), which exceeds the known measurement variability of MRE [17].

### 2.5. Statistical Analysis

Long-term changes in liver stiffness were assessed using the liver LSM ratio at the third time point relative to baseline (LSM_3_/LSM_1_). The distribution of long-term liver stiffness changes was evaluated using bar graphs and scatter plots.

Between-group differences were assessed using the Mann–Whitney U test. Univariable and multivariable logistic regression analyses were performed to evaluate whether early response was independently associated with long-term liver stiffness improvement, with baseline LSM included as a forced covariate in the multivariable model to account for potential regression to the mean. Results were reported as odds ratios with 95% confidence intervals. A two-sided *p*-value < 0.05 was considered statistically significant.

As a sensitivity analysis, multivariable logistic regression was additionally performed, adjusting for baseline liver stiffness and ≥7% body weight reduction between the first and second MRE assessments, in accordance with the Japanese guidelines for MASLD [18]. Body weight reduction was defined relative to body weight at the first MRE assessment.

Generative AI tools were used to assist in generating R code for statistical analysis. All analyses were verified and conducted by the authors.

## 3. Results

### 3.1. Patient Characteristics

Patient characteristics are shown in Table 1. A total of 100 patients were included in the final analysis. The median age was 69 years (IQR, 61–75), and 53% (*n* = 53) were male. Hypertension, diabetes mellitus, and dyslipidemia were present in 52% (*n* = 52), 60% (*n* = 60), and 36% (*n* = 36) of patients, respectively. The median baseline liver stiffness measured at the first MRE (LSM_1_) was 4.97 kPa (IQR, 3.52–6.45), and the median baseline body weight was 68.2 kg (IQR, 60.0–79.0).

A total of 22 patients were classified as early responders and 78 as non-early responders. There were no significant differences between the two groups in age, sex distribution, baseline body weight, or the prevalence of hypertension, diabetes mellitus and dyslipidemia. Baseline laboratory parameters were comparable between the two groups. Baseline liver stiffness was also similar between early responders (median, 4.97 kPa; IQR, 4.10–7.02) and non-early responders (median, 5.06 kPa; IQR, 3.27–6.22; *p* = 0.15).

When patients were categorized according to MRE-derived fibrosis thresholds reported in a previous meta-analysis, early responders were more frequently classified into categories suggestive of advanced fibrosis or cirrhosis at baseline, whereas no early responders were observed in the F0 or F2 categories (Fisher’s exact test, *p* = 0.04). The overall distribution of baseline MRE-derived fibrosis categories between early responders and non-early responders is shown in Table 1.

### 3.2. Changes in Liver Stiffness over Time

**Figure 2:**
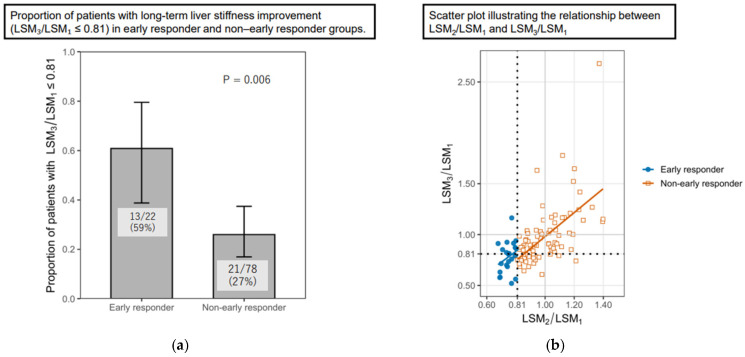
(**a**) Proportion of patients with long-term liver stiffness improvement (LSM_3_/LSM_1_ ≤ 0.81) in early-responder and non-early-responder groups. Early response was defined as a ≥19% reduction in liver stiffness at 1 year (LSM_2_/LSM_1_ ≤ 0.81). Error bars indicate 95% confidence intervals calculated using the Wilson method (early responders: 0.38–0.77; non-early responders: 0.18–0.38). Group comparisons were performed using Fisher’s exact test. (**b**) Scatter plot illustrating the relationship between LSM_2_/LSM_1_ and LSM_3_/LSM_1_. Patients with lower LSM_2_/LSM_1_ tended to have lower LSM_3_/LSM_1_ values. Dashed lines indicate the threshold corresponding to a 19% reduction in liver stiffness (LSM ratio = 0.81).

During follow-up, early responders were more likely to achieve a ≥19% reduction in liver stiffness at the third MRE (first→third MRE) compared with non-early responders (13/22 (59%) vs. 21/78 (27%), *p* = 0.006), as shown in Figure 2a. Scatter plots illustrated a modest tendency toward lower LSM_3_/LSM_1_ values among patients with lower LSM_2_/LSM_1_ (Figure 2b).

Among patients without early improvement, 21 of 78 (27%) subsequently achieved a ≥19% reduction in liver stiffness at the third MRE. Conversely, among early responders, 9 of 22 (41%) did not maintain improvement at the third MRE.

### 3.3. Factors Associated with Long-Term Liver Stiffness Improvement

In univariate analysis, lower serum albumin levels and higher baseline liver stiffness were significantly associated with long-term liver stiffness improvement (albumin: OR, 0.26; 95% CI, 0.09–0.74; *p* = 0.01; LSM_1_: OR, 1.52; 95% CI, 1.19–1.93; *p* < 0.01), whereas no significant associations were found for other clinical and biochemical variables (Table 2).

**Figure 3:**
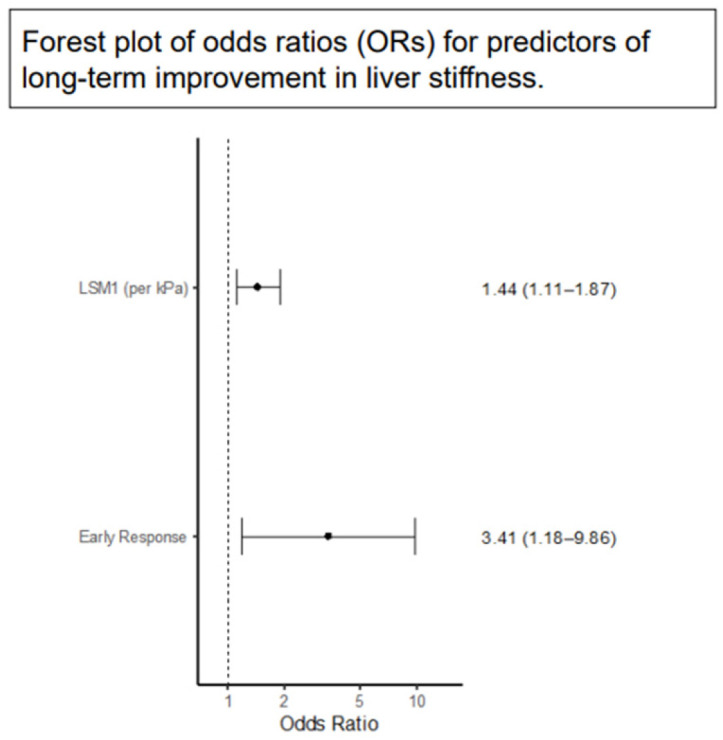
Forest plot of odds ratios (ORs) for factors associated with long-term improvement in liver stiffness. The horizontal axis is shown on a logarithmic scale with a dashed reference line at OR = 1. Points represent estimated odds ratios (ORs), and whiskers indicate 95% confidence intervals (95% CIs). Numerical ORs with corresponding 95% CIs are displayed to the right of each point. Baseline liver stiffness (LSM_1_) was associated with the outcome (OR 1.44, 95% CI 1.11–1.87), and early response showed a stronger association (OR 3.41, 95% CI 1.18–9.86).

In multivariable analysis adjusting for baseline liver stiffness, early improvement at the second measurement remained independently associated with long-term liver stiffness improvement (OR, 3.41; 95% CI, 1.18–9.86; *p* = 0.023). Higher baseline liver stiffness was also significantly associated with subsequent improvement (OR per 1 kPa increase, 1.44; 95% CI, 1.11–1.87; *p* = 0.006) (Figure 3). In contrast, serum albumin was not independently associated with long-term improvement after adjustment (OR, 0.91; 95% CI, 0.82–1.00; *p* = 0.058).

As baseline liver stiffness was independently associated with the outcome, we further explored whether the association between early response and long-term improvement varied by baseline liver stiffness. In an exploratory subgroup analysis using a baseline LSM threshold of 4.0 kPa, early response was significantly associated with long-term liver stiffness improvement in patients with baseline LSM ≥ 4.0 kPa (OR 6.19, 95% CI 1.72–22.2; *p* = 0.005). In contrast, no significant association was observed in patients with baseline LSM <4.0 kPa (OR 0.96, 95% CI 0.09–10.1; *p* = 0.97), although interpretation was limited by the small sample size and low event rate (Supplementary Table S1).

In a sensitivity analysis additionally adjusting for ≥7% body weight reduction between the first and second MRE assessments, the association between early response and long-term liver stiffness improvement remained materially unchanged. ≥7% body weight reduction between MRE_1_ and MRE_2_ was not independently associated with long-term improvement (Supplementary Figure S1).

In an additional exploratory analysis including the ALT ratio between the first and second assessments (ALT_2_/ALT_1_), a lower ALT ratio was independently associated with long-term liver stiffness improvement (Supplementary Table S2).

## 4. Discussion

### 4.1. Main Findings

The main finding of this study is that early changes in liver stiffness were associated with subsequent long-term changes in liver stiffness assessed by repeated MRE. This association remained significant after adjustment for baseline liver stiffness.

### 4.2. Interpretation of Findings

The present findings suggest that early changes in liver stiffness may reflect an underlying pattern of change that tends to persist over time. Patients who demonstrated short-term improvement were more likely to maintain or further improve liver stiffness at later time points, whereas those without early improvement were less likely to show subsequent substantial reductions. This pattern implies that early MRE changes may serve as an indicator of the direction of subsequent liver stiffness evolution rather than representing a transient fluctuation.

In addition, the association between long-term improvement and initial LSM suggests that patients with high initial LSM values may require longer-term follow-up rather than relying solely on short-term assessment. Indeed, even among patients who did not achieve short-term improvement, 27% demonstrated long-term improvement, while 41% of early responders did not maintain improvement at the third assessment. These findings suggest that changes in liver stiffness are not necessarily linear over time and may follow heterogeneous trajectories.

In an exploratory subgroup analysis, early response was significantly associated with long-term improvement in patients with higher baseline LSM, but not in those with lower baseline LSM, although the latter analysis was limited by sample size. Taken together, baseline liver stiffness and early response may offer complementary prognostic information, with early response appearing particularly useful in patients with higher baseline stiffness.

Although lower albumin levels were associated with long-term improvement in univariable analysis, this association was attenuated after adjustment for baseline liver stiffness, suggesting that the observed relationship may have been largely driven by its correlation with baseline fibrosis severity rather than an independent effect of albumin itself.

Because weight reduction is an important therapeutic target in MASLD, we additionally performed a sensitivity analysis adjusting for ≥7% body weight reduction between the first and second MRE assessments. The association between early MRE response and long-term liver stiffness improvement remained consistent, supporting the robustness of the primary findings.

Baseline ALT was not associated with long-term liver stiffness improvement. However, longitudinal reduction in ALT between the first and second assessments was independently associated with subsequent liver stiffness improvement, suggesting that dynamic changes in ALT may provide additional information compared with a single baseline ALT measurement and may reflect changes in hepatic inflammatory activity.

### 4.3. Comparison with Published Literature

Previous studies have demonstrated that liver stiffness assessed by MRE and its longitudinal changes are associated with clinical outcomes, including hepatocellular carcinoma, hepatic decompensation and mortality [14,15,19,20]. However, most of the longitudinal studies evaluated liver stiffness at two time points, typically comparing baseline and follow-up measurements over relatively short or heterogeneous intervals [14,15].

Data derived from repeated MRE assessments at three or more time points remain limited, and the relationship between early changes in liver stiffness and subsequent long-term changes in liver stiffness has not been well characterized [14,15,19,20]. In this context, our study extends existing literature by evaluating serial MRE measurements at three predefined time points, allowing assessment of how short-term changes relate to subsequent long-term changes in liver stiffness.

In addition, given the limitations of liver biopsy, including sampling variability, inter- and intra-observer variability, and limited feasibility for repeated assessment, non-invasive modalities such as MRE are increasingly utilized for longitudinal evaluation. Recent regulatory perspectives, including guidance from the U.S. Food and Drug Administration, as well as clinical studies and trials, have increasingly emphasized the use of non-invasive biomarkers and clinical outcomes rather than relying solely on histological endpoints [21]. Furthermore, previous studies have demonstrated that MRE-derived liver stiffness correlates well with fibrosis stage and predicts clinical outcomes [9,13,19,22]. These considerations support the relevance of using serial MRE measurements to assess longitudinal changes in liver disease.

### 4.4. Strengths and Limitations

A major strength of this study is the use of three sequential MRE assessments, which enabled evaluation of the association between short-term changes and longer-term changes in liver stiffness. In addition, the analysis was based on real-world clinical data, reflecting routine follow-up practices.

However, several limitations should be acknowledged. This was a single-center, retrospective study with a relatively limited sample size, which may limit generalizability and statistical power. In particular, the small number of early responders may have contributed to imprecise effect estimates and relatively wide confidence intervals. The study population likely represented a selected subgroup of patients at relatively higher clinical risk and with sufficient adherence to undergo repeated follow-up assessments, which may have introduced selection bias and limited generalizability.

Although multivariable analyses were performed, residual confounding could not be fully excluded. While body weight reduction was considered in a sensitivity analysis, other potentially relevant factors, including changes in metabolic control, lifestyle interventions, and medication use during follow-up, were not fully accounted for. Because treatment decisions were individualized in routine clinical practice, some variability in follow-up management could not be fully standardized.

In addition, the threshold of a 19% reduction in liver stiffness used to define early response was selected based on the previously reported measurement variability of MRE. However, the optimal cutoff for defining clinically meaningful early improvement remains uncertain and warrants further investigation.

Furthermore, the observed association between higher baseline liver stiffness and subsequent improvement should be interpreted with caution, as changes in liver stiffness may partly reflect a mathematical property whereby relative changes are influenced by baseline values, potentially resulting in greater apparent improvement among patients with higher initial measurements.

Another limitation is the absence of paired liver biopsy data, which limits direct histological interpretation of changes in liver stiffness. In addition, reductions in liver stiffness may reflect not only fibrosis regression but also an improvement in hepatic inflammation, as MRE-derived liver stiffness is influenced by both fibrosis and inflammatory activity [14,19]. Although ALT changes were considered in an exploratory analysis, magnetic resonance imaging proton density fat fraction (MRI-PDFF) was not routinely available; therefore, the direct impact of changes in hepatic steatosis on MRE values could not be evaluated. Baseline MRE-derived fibrosis categories were based on previously reported thresholds and were used only for descriptive characterization.

Finally, the development of hepatic decompensation and hepatocellular carcinoma are important clinical events that determine the prognosis of MASLD; however, this study did not evaluate the relationship between these clinical outcomes and liver stiffness; therefore, the prognostic significance of liver stiffness improvement remains to be determined.

### 4.5. Future Implications

Short-term changes in liver stiffness assessed by MRE may serve as an early indicator of subsequent longitudinal liver stiffness trajectories. If validated in larger prospective studies, early MRE responses could help rationalize follow-up strategies by identifying patients who may benefit from more or less intensive serial MRE monitoring. Furthermore, such early assessments may allow evaluation of treatment responsiveness without the need to await long-term outcomes, potentially facilitating earlier clinical decision-making. These findings provide a rationale for prospective studies evaluating MRE-guided follow-up strategies.

## 5. Conclusions

Short-term changes in liver stiffness measured by MRE were associated with subsequent long-term changes in patients with MASLD. These findings suggest that early assessment of liver stiffness may help identify patients who require closer monitoring and tailored management strategies.

## Figures and Tables

**Table 1:** Baseline characteristics of the study population according to early MRE response.

	Overall (*n* = 100)	Early Responder (*n* = 22)	Non-Early Responder (*n* = 78)	*p*-Value
**Age**	69 [61, 75]	71 [66, 79]	69 [59, 75]	0.11
**Male sex**	53 (53)	10 (45.5)	43 (55.1)	0.47
**Hypertension**	52 (52)	12 (54.5)	40 (51.3)	0.81
**Diabetes mellitus**	60 (60)	14 (63.6)	46 (59.0)	0.81
**Insulin use**	7 (7)	0 (0)	7 (15.2)	—
**Dyslipidemia**	36 (36.0)	9 (40.9)	27 (34.6)	0.62
**Baseline body weight (kg)**	68.2 [60.0, 79.0]	67.9 [62.7, 79.1]	68.9 [59.5, 79.0]	0.66
**Platelets (×10^4^/μL)**	15.0 [11.8, 19.9]	13.8 [11.4, 16.3]	15.6 [11.8, 20.9]	0.31
**AST (U/L)**	46 [34, 59]	45 [41, 61]	47 [33, 59]	0.56
**ALT (U/L)**	49 [31, 69]	47 [34, 67]	50 [30, 70]	0.87
**GGT (U/L)**	64 [42, 104]	60 [45, 91]	65 [41, 105]	0.76
**Albumin (g/dL)**	4.2 [3.9, 4.4]	4.1 [3.8, 4.2]	4.3 [3.9, 4.5]	0.09
**Bilirubin (mg/dL)**	0.7 [0.5, 1.0]	0.7 [0.5, 0.9]	0.7 [0.6, 1.1]	0.71
**T-Chol (mg/dL)**	184 [164, 202]	184 [164, 198]	187 [165, 205]	0.86
**HbA1c (%)**	6.7 [5.8, 7.1]	6.6 [5.7, 7.8]	6.7 [5.9, 7.1]	0.90
**LSM_1_ (kPa)**	4.97 [3.52, 6.45]	4.97 [4.10, 7.02]	5.06 [3.27, 6.22]	0.15
**Baseline LSM-derived** **fibrosis category**				0.04
**F0**	10 (10.0)	0 (0)	10 (12.8)	
**F1**	7 (7.0)	1 (4.5)	6 (7.7)	
**F2**	11 (11.0)	0 (0)	11 (14.1)	
**F3**	15 (15.0)	6 (27.3)	9 (11.5)	
**F4**	57 (57.0)	15 (68.2)	42 (53.8)	

Values are presented as median (interquartile range) or number (%). Early responders were defined as patients with ≥19% reduction in liver stiffness at 1 year (LSM_2_/LSM_1_ ≤ 0.81). ALT, alanine aminotransferase; AST, aspartate aminotransferase; GGT, γ-glutamyl transpeptidase; HbA1c, hemoglobin A1c; LSM, liver stiffness measurement; T-chol, total cholesterol. Baseline MRE-derived fibrosis categories were defined using previously reported thresholds for MASLD: F0, <2.61 kPa; F1, 2.61–2.97 kPa; F2, 2.97–3.62 kPa; F3, 3.62–4.69 kPa; and F4, ≥4.69 kPa.

**Table 2:** Univariate analysis of factors associated with improvement between MRE_1_ and MRE_3_.

	Odds Ratio	95% CI	*p*-Value
**Age**	0.99	0.97–1.03	0.95
**Male Sex**	0.83	0.36–1.91	0.67
**Hypertension**	0.89	0.39–2.03	0.77
**Diabetes mellitus**	0.77	0.33–1.79	0.55
**Dyslipidemia**	1.69	0.72–3.97	0.23
**Baseline body weight (kg)**	1.03	0.99–1.07	0.07
**≥7% body weight reduction between MRE_1_ and MRE_2_**	1.60	0.50–5.14	0.43
**Platelets (×10^4^/μL)**	0.94	0.88–1.01	0.10
**AST (U/L)**	1.00	0.98–1.02	0.98
**ALT (U/L)**	0.99	0.98–1.01	0.65
**GGT (U/L)**	1.00	0.99–1.00	0.92
**Albumin (g/dL)**	0.26	0.09–0.74	0.01
**Bilirubin (mg/dL)**	1.07	0.40–2.85	0.89
**T-Chol (mg/dL)**	1.00	0.98–1.01	0.97
**HbA1c (%)**	1.17	0.58–2.35	0.66
**LSM_1_ (kPa)**	1.52	1.19–1.93	<0.01

Odds ratios (ORs) and 95% confidence intervals (CIs) were calculated using univariable logistic regression analysis. Improvement between MRE_1_ and MRE_3_ was defined as a reduction in liver stiffness at the third examination relative to baseline (LSM_3_/LSM_1_ < 0.81). ALT, alanine aminotransferase; AST, aspartate aminotransferase; CI, confidence interval; GGT, γ-glutamyl transpeptidase; HbA1c, hemoglobin A1c; LSM, liver stiffness measurement; OR, odds ratio; T-chol, total cholesterol.

## Data Availability

The raw data supporting the conclusions of this article will be made available by the authors on request.

## Supplementary Materials

The following supporting information can be downloaded at https://www.mdpi.com/article/10.3390/diagnostics16121833/s1, Figure S1: Forest plot of odds ratios (ORs) for predictors of long-term LSM improvement; Table S1: Univariable analysis of the association between early response and long-term liver stiffness improvement according to baseline liver stiffness strata; Table S2: Multivariable analysis of factors associated with long-term liver stiffness improvement, including the ALT ratio as an exploratory variable.
