# Supplementary material for: Association Between Early and Long-Term Changes in Liver Stiffness in Patients with MASLD Undergoing Serial Magnetic Resonance Elastography
Source: Diagnostics (Basel). 2026 Jun 13;16(12):1833. doi: 10.3390/diagnostics16121833 (PMC13298384; doi:10.3390/diagnostics16121833)
Supplement: Supplementary file 1 [file diagnostics-16-01833-s001.zip › diagnostics-4259959-supplementary.pdf]

[Supplementary Information]

Supplementary Table S1 Univariable analysis of the association between early response and long-term liver stiffness improvement according to baseline liver stiffness strata

Baseline liver stiffness group	Odds Ratio	95% CI	P value
**LSM_1 <4.0 kPa**	0.96	0.09 – 10.1	0.97
**LSM_1 ≥4.0 kPa**	6.19	1.72 – 22.2	< 0.01

Early response was defined as a ≥19% reduction in liver stiffness at the 2nd examination relative to baseline ($LSM_2/LSM_1 \leq 0.81$). Long-term improvement was defined as a ≥19% reduction in liver stiffness at the 3rd examination relative to baseline ($LSM_3/LSM_1 \leq 0.81$). Odds ratios were estimated using univariable logistic regression analysis within each subgroup. CI, confidence interval; LSM, liver stiffness measurement; MRE, magnetic resonance elastography.

Supplementary Table S2 Multivariable analysis of factors associated with long-term liver stiffness improvement, including ALT ratio as an exploratory variable

	Odds Ratio	95% CI	P value
**Baseline LSM**	1.49	1.15 – 1.93	<0.01
**Early Response**	2.34	0.78 – 7.05	0.13
**ALT ratio (ALT_2/ALT_1)**	0.16	0.03 – 0.77	0.02

Early response was defined as $LSM_2/LSM_1 \leq 0.81$, and long-term improvement as $LSM_3/LSM_1 \leq 0.81$. ALT ratio was defined as ALT_2/ALT_1. Odds ratios were estimated using multivariable logistic regression analysis. ALT, alanine aminotransferase; CI, confidence interval; LSM, liver stiffness measurement.

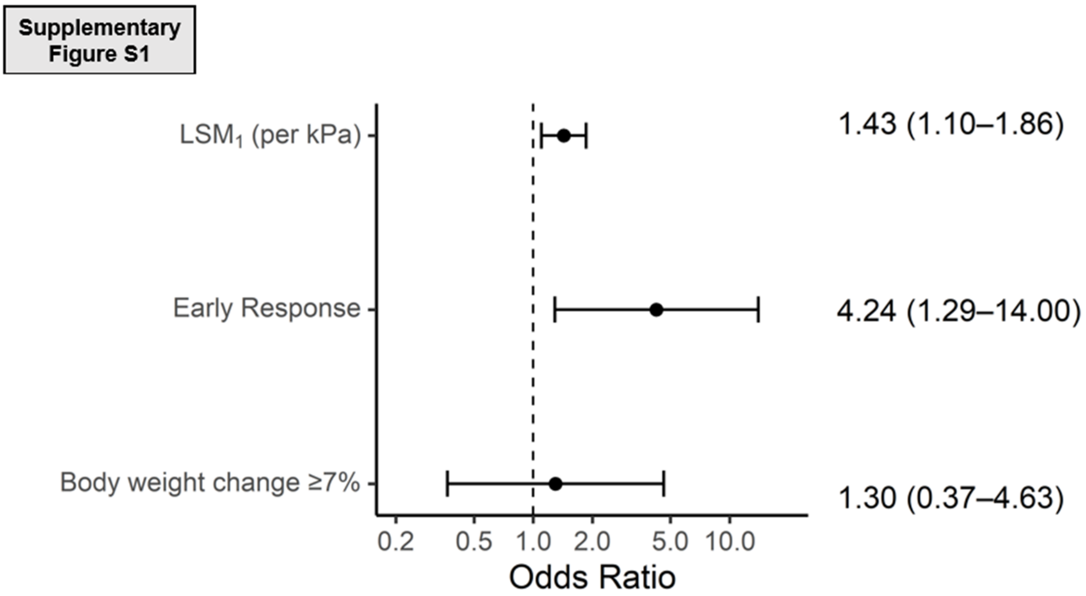

Supplementary Figure S1 Forest plot of odds ratios (ORs) for predictors of long-term LSM improvement.

In contrast to the primary analysis shown in Figure 3, this model additionally includes body weight change (≥7%) as a covariate to evaluate its potential contribution to the prediction of response. Odds ratios are presented with 95% confidence intervals (CIs). The dashed vertical line indicates an OR of 1 (no association).
